# Exposure–response modelling approaches for determining optimal dosing rules in children

**DOI:** 10.1177/0962280220903751

**Published:** 2020-02-13

**Authors:** Ian Wadsworth, Lisa V Hampson, Björn Bornkamp, Thomas Jaki

**Affiliations:** 1 Department of Mathematics & Statistics, Fylde College, Lancaster University, Lancaster, UK; 2 Phastar, Macclesfield, UK; 3 Advanced Methodology & Data Science, Novartis Pharma AG, Basel, Switzerland

**Keywords:** Bayesian penalised B-splines, dosing rules, exposure–response modelling, model-based recursive partitioning, paediatric

## Abstract

Within paediatric populations, there may be distinct age groups characterised by different exposure–response relationships. Several regulatory guidance documents have suggested general age groupings. However, it is not clear whether these categorisations will be suitable for all new medicines and in all disease areas. We consider two model-based approaches to quantify how exposure–response model parameters vary over a continuum of ages: Bayesian penalised B-splines and model-based recursive partitioning. We propose an approach for deriving an optimal dosing rule given an estimate of how exposure–response model parameters vary with age. Methods are initially developed for a linear exposure–response model. We perform a simulation study to systematically evaluate how well the various approaches estimate linear exposure–response model parameters and the accuracy of recommended dosing rules. Simulation scenarios are motivated by an application to epilepsy drug development. Results suggest that both bootstrapped model-based recursive partitioning and Bayesian penalised B-splines can estimate underlying changes in linear exposure–response model parameters as well as (and in many scenarios, better than) a comparator linear model adjusting for a categorical age covariate with levels following International Conference on Harmonisation E11 groupings. Furthermore, the Bayesian penalised B-splines approach consistently estimates the intercept and slope more accurately than the bootstrapped model-based recursive partitioning. Finally, approaches are extended to estimate Emax exposure–response models and are illustrated with an example motivated by an in vitro study of cyclosporine.

## 1 Introduction

Children of different ages given a new medicine may be characterised by different dose–exposure and exposure–response (E–R) relationships due to age-related differences in growth, development and physiological differences.^1^ Several regulatory guidance documents have suggested general age groupings, such as the International Conference on Harmonisation (ICH) E11 document,^1^ which suggests one possible categorisation: preterm newborn infants; term newborn infants (0–27 days); infants and toddlers (28 days to 23 months); children (2–11 years); and adolescents (12 to 16–18 years, depending on region). The National Institute of Child Health and Human Development (NICHD) guideline, suggests similar age groups, but with extra splits at 1 and 6 years. This paper aims to estimate the E–R relationship in children and to identify age groupings which define practical and effective dosing rules.

An understanding of how the E–R relationship of a drug varies with age will inform whether and how we leverage adult data to support drug development in children. Hampson et al.^2^ reviewed paediatric investigation plans (PIPs) and found that it was common to plan to identify paediatric doses by matching target adult exposures. This is an appropriate dose-finding strategy if E–R relationships are similar in adults and children. This assumption might be justified for some paediatric subgroups but not others. For example, Takahashi et al.^3^ concluded that whilst pubertal (12–18 years) and adult patients had similar PD responses to long-term warfarin therapy, there were differences between pre-pubertal (1–11 years) patients versus pubertal and adult patients. If E–R relationships can be assumed to be similar across age groups, it may be appropriate to make a complete extrapolation of efficacy data from one age group to another, so that only dose–exposure data are needed in the unstudied age group to identify doses producing exposures known to be efficacious in the studied age group.^2,4^ However, if E–R relationships cannot be considered similar, a partial extrapolation approach^4^ may be considered, where dose–exposure and E–R data may be accrued in specified age groups to confirm differences in E–R relationships and confirm dosing.

One common approach to modelling nonlinear E–R relationships is the Emax model.^5^ Thomas et al.^6^ show that the Emax model provides good fit to the dose–response relationship of almost all compounds and diseases in the time window they studied. Parkinson et al.^7^ developed a sigmoid Emax model for the relationship between dapagliflozin exposure and urinary glucose excretion for adult and paediatric patients with type 2 diabetes mellitus. After accounting for significant covariates (e.g. sex, race, baseline fasting plasma glucose), further covariates were included for paediatric patients which failed to improve model fit. The authors took this as evidence that adult and paediatric patients had similar E–R relationships. Earp et al.^8^ used E–R modelling and exposure matching analyses to estimate paediatric doses for esomeprazole for the treatment of gastroesophageal reflux disease. The authors modelled E–R relationships of intragastric pH for adults and children separately and concluded similarity of E–R based on a visual inspection of fitted E–R relationships. In this paper, a more quantitative approach to evaluating differences between E–R relationships is taken using sophisticated modelling approaches.

Age groups characterised by different E–R relationships can be considered as distinct subgroups. Lipkovich et al.^9^ reviewed methods for the identification and analysis of subgroups in clinical trials. Ondra et al.^10^ reviewed methods for designing and analysing clinical trials that aim to investigate differences in treatment effects across subgroups. In this paper, we consider two model-based approaches to quantifying how E–R model parameters vary over a continuous age range: Bayesian penalised B-splines,^11^ and model-based recursive partitioning (MOB)^12,13^ which is used to fit model-based trees to bootstrapped samples of the E–R data. Based on estimates of how E–R model parameters vary with age, we propose an approach to identify the age groups and exposure levels that define a dosing rule which is optimal for targeting a certain level of response; definition of the dosing rule is then completed by using the exposure levels and estimated dose–exposure relationship to make dosing recommendations for each age group. The estimated dose–exposure relationship is not considered in this paper.

Thomas et al.^14^ use MOB to estimate patient subgroups with different dose–response curves, and apply this method to data from a dose-finding trial. In this paper, we focus on estimating age groups with different E–R relationships since in practice, when seeking to relate dose to response, a two-step process relating dose to exposure then exposure to response is often adopted. For example, the ICH E4 guidance^15^ states that E–R information can help to identify a range of concentrations likely to lead to a satisfactory response, which can in turn inform dose selection. While parameters of the dose–exposure relationship are expected to depend on age, for some medicines, parameters of the E–R relationship are expected to remain stable across age groups. In such cases, the two-step modelling process can be advantageous because it enables separate modelling of the dose–exposure and E–R relationships, which allows for changes due to age to be captured in each relationship separately. In a simulation study to compare the performance of the two-step and single-stage approaches to dose finding, Berges and Chen^16^ found that the two-step approach resulted in more precise E–R model parameter estimation and more accurate dose selection, although gains depend on properties of the drug, trial design features and the target response.

Pharmacokinetics (PK) is the study of the time course of drug levels in the body and the mathematical modelling of such data.^17^ Population-PK models are an extension of PK modelling, studying PK at the population level and modelling data from all individuals simultaneously.^18^ Hsu^19^ found that in scenarios with increased intrinsic PK variability, E–R modelling has advantages for dose selection over dose–response modelling, provided measurement error for exposures is small. As an example of a two-stage approach to selecting a dosing rule, Schoemaker et al.^20^ developed a population PK model to describe the relationship between brivaracetam dose and plasma concentration in adults with partial onset seizures, and a population PK-pharmacodynamic model to describe the relationship between brivaracetam plasma concentration and daily seizure counts. The authors then simulated from these models to estimate the relationship between dose and response, enabling them to identify a dose range producing the maximum response.

This paper proceeds as follows. Section 2 gives a motivating example while Section 3 defines two E–R models. In Section 4, we introduce the methods that will be used to estimate parameters of E–R relationships. Section 5 proposes an approach for using fitted E–R models to identify practical dosing rules for children. We use simulation to evaluate the performance of E–R modelling approaches and the operating characteristics of the dosing rule algorithm. The design of the simulation study is described in Section 6, and the results are presented in Section 7. An example illustrating how the E–R modelling approaches can be applied to non-linear models is given in Section 8. The paper concludes with a discussion in Section 9.

## 2 Motivating example

We motivate the work that follows by considering the development of epilepsy medicines for paediatric patients with partial onset seizures. Girgis et al.^21^ study both monotherapy and adjunctive therapy with the anti-epileptic drug topiramate, whilst Nedelman et al.^22^ consider adjunctive therapy with oxcarbazepine. For adjunctive therapy, Girgis et al.^21^ and Nedelman et al.^22^ take response, Y=log{Z+110} , to be the log-transformed percent change from baseline in seizure frequency, where *Z* is the percent change from baseline in seizure frequency. The response, *Y*, is assumed to be normally distributed and a linear function of exposure, measured by the average steady-state trough concentration (*C*_min_). Girgis et al.^21^ and Nedelman et al.^22^ evaluate the similarity of E–R relationships in adults and children on adjunctive therapy with the aim of justifying the use of extrapolation to support the approval of monotherapy in children. The models and the parameter estimates^21^ will be used to inform the design of realistic simulation scenarios.

## 3 Exposure–response models

We start by considering a linear model for the E–R relationship. Suppose E–R data are available from a single study which recruited children aged 0 to 18 years and let *Y_i_* represent the response of subject *i*, for i=1,…,N . If the E–R relationship does not depend upon age, we could model it as

$$Y_{i}=\gamma_{0}+\sum_{p=1}^{P}\gamma_{p}x_{pi}+\gamma_{C}C_{i}+\epsilon_{i}$$

where *C_i_* is a measure of drug exposure (such as *C*_min_), $x_{1i},\ldots ,x_{Pi}$ are other covariates influencing response (such as body weight), and $\epsilon_{i}∼N(0,\sigma^{2})$ is a random error term. We consider the situation where the E–R relationship may differ between age groups, that is, *γ*_0_ and *γ_C_* are functions of age (*A*)

(1) $$Y_{i}=\gamma_{0}(A_{i})+\sum_{p=1}^{P}\gamma_{p}x_{pi}+\gamma_{C}(A_{i})C_{i}+\epsilon_{i}$$

In Section 4 we will consider different approaches for parameterising $\gamma_{0}(A_{i})$ and $\gamma_{C}(A_{i})$ .

Non-linear Emax models are often used to represent the E–R relationship.^23^ For example, it could be modelled by a sigmoid Emax model:

(2) $$Y_{i}=\gamma_{0}(A_{i})+\sum_{p=1}^{P}\gamma_{p}x_{pi}+\frac{E_{\max}(A_{i})C_{i}^{\delta (A_{i})}}{EC_{50}{\left(A_{i}\right)}^{\delta (A_{i})}+C_{i}^{\delta (A_{i})}}+\epsilon_{i}$$

where for subject *i*, aged *A_i_* years old, $\gamma_{0}(A_{i})$ is the intercept, $E_{\max}(A_{i})$ is the maximum effect attributable to the drug, $EC_{50}(A_{i})$ is the concentration of the drug that produces half of the maximum effect, and $\delta (A_{i})$ (the Hill parameter) governs slope steepness. Here, four of the model parameters may potentially depend upon age.

## 4 Estimating the exposure–response relationship

In this section, we describe three E–R modelling approaches that can be applied when we assume the E–R relationship follows model (1) with age-dependent intercept and slope. These methods are linear regression with categorical covariates for age groups; MOB and partially additive linear model (PALM) trees; and Bayesian penalised B-splines. We highlight where methods can be applied more generally with non-linear E–R models. A worked example illustrating how each method can be applied to fit a linear E–R model is given in Supplementary Appendix A.

### 4.1 Linear model fit with categorical age covariates

If we knew that the age groups defined by different E–R relationships were $\left(a_{0}=0,a_{1}),(a_{1},a_{2}),\ldots ,(a_{H-1},a_{H}=18\right)$ , we could define a linear model for the E–R relationship as follows

(3) $$Y_{i}=\gamma_{0}+\sum_{p=1}^{P}\gamma_{p}x_{pi}+\gamma_{C}C_{i}+\sum_{h=2}^{H}I_{A_{h}}(A_{i})\left\{\gamma_{A,h}+\gamma_{I,h}C_{i}\right\}+\epsilon_{i}$$

where $A_{h}$ is the interval $\left(a_{h-1},a_{h}\right)$ ; $I_{A_{h}}(A_{i})$ is an indicator function (1 if $A_{i}\in A_{h}$ , 0 otherwise); $\gamma_{A,2},\ldots ,\gamma_{A,H}$ are the main effects of the age groups; and $\gamma_{I,2},\ldots ,\gamma_{I,H}$ are the interactions between age group and exposure. Fitting this model permits estimation of age group-specific intercepts and slopes. We include this simple model as a benchmark for comparison with other more complex modelling approaches. Unlike the other methods we consider, this approach requires that age groups be pre-specified rather than estimating them from the data.

### 4.2 MOB and PALM trees

Building on model (3), MOB allows data to be split into groups based on partitioning variables, with each subgroup characterised by its own parametric model.^13^ We implement MOB using age as the only partitioning variable. The MOB algorithm we use comprises the following steps^13^: Fit a parametric model to the data set, finding parameter estimates by minimising an objective function; test whether the model parameters significantly change with age using a generalized M-fluctuation test^13,24^ which assesses whether the scores of the model systematically deviate from 0 with age; partition the model into two subgroups with respect to age by finding the value of age which minimises an objective function segmented at this split point; repeat the fitting, testing and splitting procedure in each identified age group until no significant changes are found in the model parameters over age within each group. In our subsequent examples, the parametric model will be taken to be a linear model, where the parameters of interest are the intercept and slope. The MOB algorithm^13^ can be implemented using the ‘mob’ function found in the ‘partykit’ package^13,25^ in R.^26^ As MOB allows subgroups defined by any parametric model, non-linear models (such as Emax models) are possible.

PALM trees are a variation of MOB, allowing for global parameters which remain constant across subgroups. However, PALM trees are restricted to generalised linear models (GLM).^12^ For our linear model example with outcome *Y_i_* and partitioning age variable *A_i_*, PALM trees can contain globally fixed linear effects $\gamma_{1},\ldots ,\gamma_{P}$ for covariates $x_{1i},\ldots ,x_{Pi}$ and subgroup-wise varying linear effects $\gamma_{0}(A_{i})$ and $\gamma_{C}(A_{i})$ , as in equation (1). PALM trees use the MOB algorithm described above to identify age groups with distinct GLMs. In order to allow for global parameters which remain constant across age groups, an EM-type algorithm is used. This iterates between estimating the global effects, $\gamma_{1},\ldots ,\gamma_{P}$ , for the current PALM tree and estimating the PALM tree (using the above MOB algorithm) for a given set of global effect estimates, ${\hat{\gamma }}_{1},\ldots ,{\hat{\gamma }}_{P}$ . The algorithm can be implemented in R^26^ using the ‘palmtree’ function found in the ‘partykit’ package.^12,25^ We implement PALM trees with the default tuning parameters, i.e. a significance level of 0.05 and no maximum tree depth. An advantage of tree-based methods is the easy to understand output: each final partitioned subgroup of the tree represents an age group, with model parameter estimates given for each group.

We implement MOB and PALM tree approaches using bootstrap aggregating^27^ to improve the accuracy and precision of age-specific E–R model parameter estimates and reduce overfitting. The E–R data are bootstrapped and each bootstrap sample is used to fit a MOB or PALM tree. From each bootstrap tree fit, estimates of age-specific model parameters (intercept and slope) can be evaluated for a grid of ages covering the interval [0, 18] years. For each grid point in turn, we then aggregate across the bootstrap samples and, applying linear interpolation to the average age-specific parameter estimates, can thus obtain an estimate of the E–R intercept or slope for any given age. The important aspect to note here is that no parametric assumptions are made about the form of the relationship between each model parameter and age. One disadvantage of this is that these relationships cannot then be easily recorded in a closed form for future reference.

We fit linear E–R models using PALM trees in Section 6 because we also consider the case of having an additional global covariate whose effect is independent of age, which we present in Supplementary Appendix B. In Section 8, we fit non-linear E–R models using MOB.

### 4.3 Bayesian penalised B-splines

Splines define flexible regression models by joining smooth curves (differentiable at every point) together at knot points.^28^ An E–R model parameter that can be written as a smooth function of *A*, *f*(*A*), can be modelled as a spline. Here, we will consider the penalised B-splines developed by Eilers and Marx.^11^ B-splines can be written as a linear combination of B-spline basis functions of degree *d*, that is, $B_{1}(A;d),\ldots ,B_{J}(A;d)$ :

(4) $$f(A)=\sum_{j=1}^{J}\beta_{j}B_{j}(A;d)$$

A B-spline basis function of degree *d* consists of *d *+* *1 polynomial curves of degree *d*, each joined in sequence.^11^ The degree of the B-spline basis controls how differentiable the spline is and can influence the smoothness of the spline. We implement B-splines of degree 2 as in the examples we have considered we gain little in terms of smoothness for the added complexity of using degree 3 B-splines. We therefore fit linear E–R models defining the intercept and slope as B-splines of degree 2

$$\begin{array}{l} \gamma_{0}(A_{i})=\sum_{j=1}^{J}\beta_{0j}B_{j}(A_{i};d=2)\\ \gamma_{C}(A_{i})=\sum_{j=1}^{J}\beta_{Cj}B_{j}(A_{i};d=2) \end{array}$$

We set *J *=* *26 given our choice of degree and number of knots: five equally spaced knots within each of the four ICH E11 age groups (not including pre-term newborn infants), knots at each age group boundary, along with two external knots below age zero and two above 18 years. We use the function ‘splineDesign’ in the R package ‘splines’^26^ to construct our 26 B-spline basis functions. Further details of how the B-spline basis functions are constructed can be found in Bowman and Evers.^28^ Note that for penalised B-splines, Eilers and Marx^11^ recommend using equidistant knots and suggest that there are no gains to be made from using unequally spaced knots, as the penalty smooths any sparse areas. However, we specify knots using the prior information on potential age groupings that is contained in the ICH E11 guidance document.^1^ By specifying an equal number of knot points across each ICH E11 age group, knots are more densely spread across age ranges where model parameters are expected to change most rapidly with age. A sensitivity analysis to explore the impact of knot placement would be appropriate in many cases.

For penalised B-splines, a roughness penalty is used to control the smoothness of the estimated spline, rather than the choice of knot location and number.^11^ In a Bayesian context, penalised B-splines are implemented placing random walk priors on the B-spline coefficients.^28,29^ For example, to penalise differences between adjacent B-spline coefficients, first-order random walk priors are used

$$\begin{array}{l} \beta_{0,j}|\beta_{0,j-1}∼N(\beta_{0,j-1},\tau_{0}^{2}),\;\text{ for }\;j=2,\ldots ,J\\ \beta_{C,j}|\beta_{C,j-1}∼N(\beta_{C,j-1},\tau_{C}^{2}) \end{array}$$

with $\beta_{0,1}∼N(0,100)$ and $\beta_{C,1}∼N(0,100)$ . This penalises B-spline coefficients by shrinking towards a common constant,^28^ which is desirable in our context since we anticipate that there may be age ranges on which a model parameter is fairly stable followed by periods of rapid change. We stipulate diffuse Inverse-Gamma (1, 0.005) priors for *τ*_0_ and *τ_C_*, similar to Lang and Brezger^29^ who place an Inverse-Gamma prior on the variance of the random walk prior. We do not weight $\tau_{0}^{2}$ and $\tau_{C}^{2}$ by the distance between successive knot points, as suggested by Kneib et al.,^30^ to allow larger prior variation when there are larger steps between knots. This is because in our setting, we have purposefully placed knots closer together over age intervals where the most rapid changes with age are anticipated.

We fit the Bayesian penalised B-splines model using Hamiltonian Monte Carlo, calling Stan^31^ from R^26^ using the RStan package,^32^ and running three chains with a default thinning rate of one for 3000 iterations, 1500 of which are discarded as burn-in samples. Following equation (4), the posterior means of the B-spline coefficients are multiplied by the B-spline basis functions to estimate the B-spline for the respective E–R model parameter.

Bayesian penalised B-splines are a very flexible modelling approach, with the capacity to be used to represent the parameters of any parametric E–R model. The ability to write the relationship between E–R parameters and age in a simple form, as in equation (4), means it is easy to record and communicate the estimated relationship. However, Bayesian penalised B-spline models can comprise many parameters which can make them computationally expensive to fit.

## 5 Dosing recommendations

### 5.1 Optimisation criterion

We could use the modelling approaches described in Section 4 to derive personalised dosing recommendations tailored to a patient’s exact age and baseline covariates. However, for practical reasons, we seek to identify dosing rules based on wider age subgroups. As outlined in Section 1, we focus on identifying age groups and exposure levels targeting a certain level of response, assuming that in a second step we could use a PK model to link each target exposure to dose. Therefore, we use ‘dosing rule’ as a short-hand to refer to a set of age groupings and corresponding target exposures. First, we derive target exposure levels for up to *K* age groups of children. For practical reasons, *K* would likely be small, e.g. *K *=* *5 in the ICH E11 guideline.^1^ When defining the target exposure for each age group, we would like to minimise the difference between the expected response and a target response denoted by $Y^{*}$ . For the epilepsy example, a 50% change in seizure frequency from baseline would be an appropriate target response, so that $Y^{*}=\log(-50+110)$ .

We derive dosing rules assuming the E–R model and parameter estimates (maximum likelihood for the frequentist approaches, posterior means for the Bayesian penalised B-splines) are identical to the true model and parameter values. Given a proposed age grouping, let *C_k_* denote the target exposure for the *k*th age group $\left(a_{k-1},a_{k}\right)$ needed for a patient aged $(a_{k-1}+a_{k})/2$ years to have expected PD response equal to $Y^{*}$ . Furthermore, define $D_{a}=|E[Y\;|\;A=a,C=C_{k}]-Y^{*}|$ . If the E–R model adjusts for a set of baseline covariates, expectations of *Y* are calculated conditioning on average covariate values at age *A *=* a*, while *C_k_* is calculated for a patient with average covariate values at age $(a_{k-1}+a_{k})/2$ . One approach would be to find the dosing rule minimising the objective function $F=\int_{0}^{18}D_{a}\;da$ , where rules minimising *F* minimise the total absolute difference between the expected response and $Y^{*}$ . *F* weights equally the performance of the dosing rule at every age. This is undesirable in our context since if E–R model parameters do depend on age, it may be reasonable to expect parameters to change rapidly over short intervals (i.e. between 0 and 2 years) and remain fairly stable across the adolescent age range. Minimising *F* would favour rules which dose most ages effectively, where inaccurate dosing over narrow age intervals would not be seriously penalised. However, our aim is to ensure all ages are dosed appropriately. With this in mind, we choose dosing rules to minimise:

(5) $$G=\frac{1}{a_{1}^{*}}\int_{0}^{a_{1}^{*}}D_{a}\text{d}a\;+\;\frac{1}{a_{2}^{*}-a_{1}^{*}}\int_{a_{1}^{*}}^{a_{2}^{*}}D_{a}\text{d}a\;+\;\ldots \;+\;\frac{1}{a_{P}^{*}-a_{P-1}^{*}}\int_{a_{P-1}^{*}}^{a_{P}^{*}}D_{a}\;\text{d}a$$

where $a_{1}^{*}<a_{2}^{*}<\ldots <a_{P}^{*}$ are fixed and pre-specified age boundaries and may be based on regulatory guidance, such as the ICH E11 guideline^1^ or the NICHD guideline.^33^ We define these boundaries in line with the NICHD guidelines. Finding dosing rules which minimise *G* means that we give equal weight to the performance of the dosing rule in a number of paediatric age groups considered as our best prior guesses.

### 5.2 Identifying an optimal number of age groups in our dosing rule

Define $\Lambda_{K}=(a_{0},\ldots ,a_{K})$ as the vector of age boundaries defining the optimal dosing rule with *K* groups; $C_{K}$ as the vector of target exposures; and $G_{K}^{*}$ as the minima of *G* for *K* age groups. Furthermore, let *K*_max_ denote the maximum number of age groups considered to be plausible or workable in practice, which would be pre-specified based on feedback from clinicians. We use the following algorithm to define a paediatric dosing rule:

1. Begin with *K* = 1 age group;
2. For *K* age groups, search over configurations of Λ*_K_* to find the dosing rule minimising *G_K_*;
3. Save $G_{K}^{*},\;\Lambda_{K}^{*}$ and $C_{K}^{*}$ ;
4. Repeat steps (2) and (3), successively increasing *K* by one until $K=K_{\max}$ .

The minima $G_{1}^{*},\ldots ,G_{K_{\max}}^{*}$ can be compared to see if increasing *K* always produces a worthwhile increase in the accuracy of the dosing rule. The optimum value of *K*, balancing the trade-off between complexity and accuracy, is denoted by $K^{*}$ . In some scenarios, a more automated approach to selecting $K^{*}$ is possible. In these cases, for each K=1,…,5 , we propose calculating the percentage difference between $G_{K+1}^{*}$ and $G_{K}^{*}$ . The value of *K* where the percentage change is less than c=25% is taken as $K^{*}$ . The arbitrary choice of *c* used here is intended to illustrate one possible approach and will be adopted in the simulation study described in the next section.

## 6 Design of the simulation study

We performed a simulation study to explore the performance of the modelling approaches described in Section 4 and the approach of Section 5 for defining dosing rules. We consider a range of data generation scenarios for the linear model described in Section 3. For the categorical age covariates model, we follow the ICH E11 age groups to fix the age intervals as $A_{1}=(0,28/365),A_{2}=(28/365,2),A_{3}=(2,12)$ and $A_{4}=(12,18)$ in equation (3), across all scenarios.

**Figure 1.**
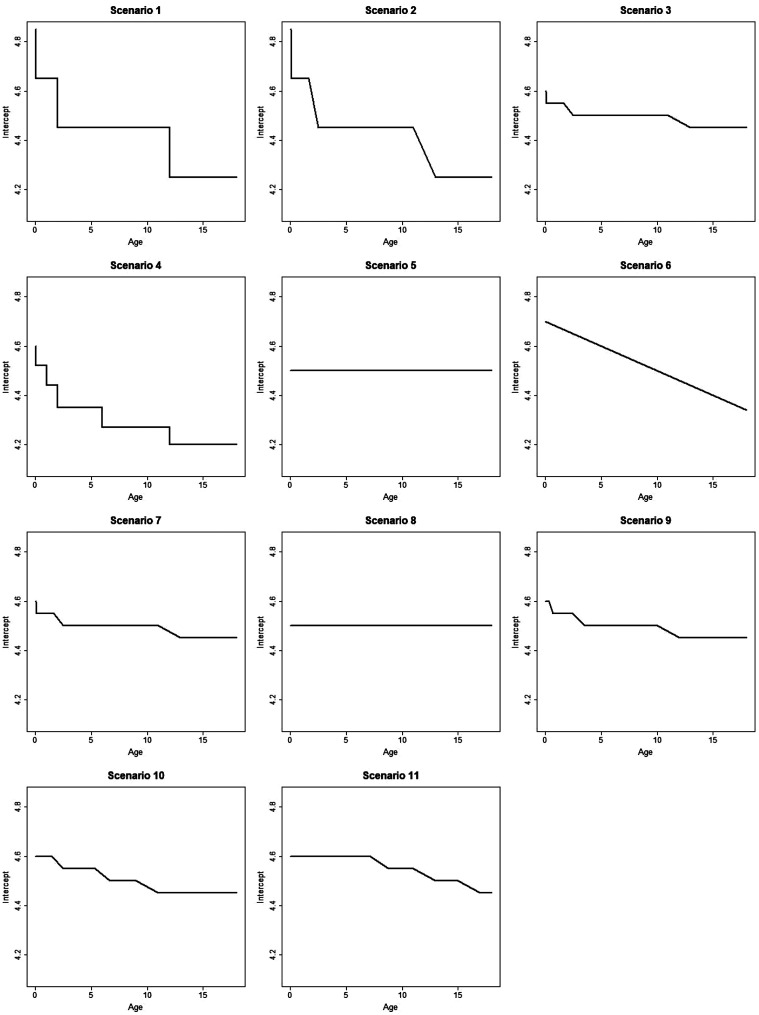
Plot showing how the intercept of the E–R model changes with age in simulation scenarios 1–11.

**Figure 2.**
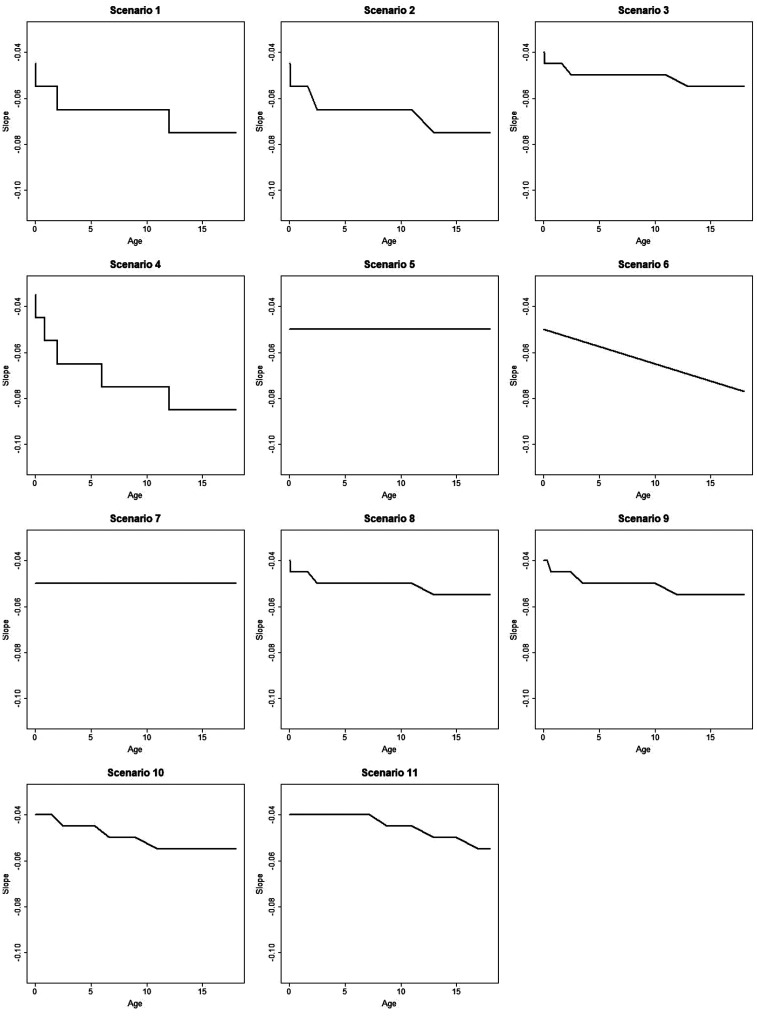
Plot showing how the slope of E–R model changes with age in simulation scenarios 1–11.

We simulate studies enrolling 25 subjects into each of four ICH E11 age groups, (0,28/365),(28/365,2),(2,12),(12,18) , excluding preterm newborn infants. Within age group $\left(a_{i-1},a_{i}\right)$ , the age of patient *i* is sampled from a Uniform $\left(a_{i-1},a_{i}\right)$ distribution. We consider 11 scenarios, as illustrated in Figures 1 and 2, for how E–R model parameters vary with age. More detail on these scenarios is provided in Supplementary Table S1, Supplementary Figure S9 and Appendix 1. We only consider scenarios where the E–R intercept and slope change monotonically with age, since these differences are most realistic in the context of the epilepsy example.

We measure exposure by *C*_min_. Following Wadsworth et al.,^34^ we sample $\log(C_{\min})$ from a N(log(2.94),0.921) distribution, truncating samples above by log(17.27) to avoid excessively high concentrations. We sample random errors from a N(0,0.02) distribution. These simulated values are used to generate patient responses, *Y_i_*, according to equation (1). We simulate 1000 data sets for each scenario and approach using the statistical software R.^26^

### 6.1 Evaluating different approaches to modelling the E–R relationship

We use the following measures to compare the modelling approaches. Define A as a grid of *Q *=* *40,000 equally spaced ages between 0 and 18 years. For each age, $A_{q}\in A$ , we first measure how well each of the methods has estimated the true intercept and slope parameters. We do this by comparing the true parameters, $\gamma_{0}(A_{q})$ and $\gamma_{C}(A_{q})$ , with our estimates of the parameters, ${\hat{\gamma }}_{0}^{\left(m\right)}(A_{q})$ and ${\hat{\gamma }}_{C}^{\left(m\right)}(A_{q})$ , based on simulated data set *m*, for m=1,…,1000 . For simplicity, henceforth we will refer to a general E–R model parameter $\gamma^{\left(m\right)}(A_{q})$ and corresponding estimate ${\hat{\gamma }}^{\left(m\right)}(A_{q})$ .

Let $\hat{E[\hat{\gamma }(A_{q})]}=\frac{1}{M}\Sigma_{m=1}^{M}{\hat{\gamma }}^{\left(m\right)}(A_{q})$ . We compute the average absolute bias (AAB), Empirical Standard Deviation (ESD) and Empirical Mean Squared Error (EMSE) of a parameter estimator at age *A_q_* as

$$\begin{array}{l} \text{AAB}\left(\hat{\gamma }\left(A_{q}\right)\right)=\frac{1}{M}\sum_{m=1}^{M}\left|{\hat{\gamma }}^{\left(m\right)}\left(A_{q}\right)-\gamma \left(A_{q}\right)\right|\\ \text{ESD}\left(\hat{\gamma }\left(A_{q}\right)\right)=\sqrt{\frac{1}{M-1}\sum_{m=1}^{M}{\left({\hat{\gamma }}^{\left(m\right)}\left(A_{q}\right)-\hat{E\left[\hat{\gamma }\left(A_{q}\right)\right]}\right)}^{2}}\\ \text{EMSE}\left(\hat{\gamma }\left(A_{q}\right)\right)=\frac{1}{M}\sum_{m=1}^{M}{\left({\hat{\gamma }}^{\left(m\right)}\left(A_{q}\right)-\gamma \left(A_{q}\right)\right)}^{2} \end{array}$$

for q=1,…,Q . Using the grids of AAB, ESD and EMSE values thus produced, we use Simpson’s rule^35,36^ to calculate the integrated absolute bias, integrated empirical SD and integrated empirical MSE for the E–R parameter estimator. These metrics can be interpreted as overall measures of accuracy, precision and MSE of an estimate of the functional relationship between an E–R model parameter and age.

Similarly, let *Y_qj_* denote the response at age, *A_q_*, and exposure, $C_{j}\in C$ , where C is a grid of *J *=* *40,000 equally spaced exposures between 0 and 18. We wish to compare the estimated expected response at exposure level *C_j_*, ${\hat{E}}^{\left(m\right)}[Y_{qj}]={\hat{\gamma }}_{0}^{\left(m\right)}(A_{q})+{\hat{\gamma }}_{C}^{\left(m\right)}(A_{q})C_{j}$ , with the true expected response at *C_j_* given by $E[Y_{qj}]=\gamma_{0}(A_{q})+\gamma_{C}(A_{q})C_{j}$ .

Let $\hat{E[Y_{qj}]}=\frac{1}{M}\sum_{m=1}^{M}{\hat{E}}^{\left(m\right)}[Y_{qj}]$ . For each j=1,…,J , and q=1,…,Q calculate

$$\begin{array}{l} \text{AAB}\left(\hat{E}\left[Y_{qj}\right]\right)=\frac{1}{M}\sum_{m=1}^{M}\left|{\hat{E}}^{\left(m\right)}\left[Y_{qj}\right]-E\left[Y_{qj}\right]\right|\\ \text{ESD}\left(\hat{E}\left[Y_{qj}\right]\right)=\sqrt{\frac{1}{M-1}\sum_{m=1}^{M}{\left({\hat{E}}^{\left(m\right)}\left[Y_{qj}\right]-\hat{E\left[Y_{qj}\right]}\right)}^{2}}\\ \text{EMSE}\left(\hat{E}\left[Y_{qj}\right]\right)=\frac{1}{M}\sum_{m=1}^{M}{\left({\hat{E}}^{\left(m\right)}\left[Y_{qj}\right]-E\left[Y_{qj}\right]\right)}^{2} \end{array}$$

These evaluations produce *Q *×* J* matrices of values for AAB, ESD and EMSE. For each *C_j_*, for j=1,…,J , we then numerically integrate over age using Simpson’s rule, and then apply Simpson’s rule again to integrate over exposure to obtain the integrated absolute bias, integrated empirical SD and integrated empirical MSE for a patient’s expected response. These can be interpreted as overall measures of the accuracy, precision and MSE of our estimate of the E–R relationship across a continuum of ages.

### 6.2 Measuring the accuracy of dosing rules

Following the algorithm of Section 5, we find dosing rules comprising K=1,…,6 age groups, with associated target exposures and minimum objective function values. We want to assess the performance of this dosing rule identification process. For the mth simulated data set, we first take the derived *K* ‘optimal’ age groups, $\left(a_{0}^{\left(m\right)}=0,a_{1}^{\left(m\right)}),\ldots ,(a_{K-1}^{\left(m\right)},a_{K}^{\left(m\right)}=18\right)$ , and estimates of corresponding target exposure levels, ${\hat{C}}_{1}^{\left(m\right)},\ldots ,{\hat{C}}_{K}^{\left(m\right)}$ , and evaluate the true expected response, at the target exposure levels, according to the simulation model. That is, at age $A_{q}\in A$ , we define

$${\hat{E}}^{\left(m\right)}\left[Y_{qK}\right]=\sum_{k=1}^{K}I_{A_{k}^{\left(m\right)}}\left(A_{q}\right)\left[\gamma_{0}\left(A_{q}\right)+\gamma_{C}\left(A_{q}\right){\hat{C}}_{k}^{\left(m\right)}\right]$$

for q=1,…,Q, where $A_{k}^{\left(m\right)}$ is the interval $\left(a_{k-1}^{\left(m\right)},a_{k}^{\left(m\right)}\right)$ and $I_{A_{k}^{\left(m\right)}}(A_{q})$ is an indicator function, which takes the value 1 if $A_{q}\in A_{k}^{\left(m\right)}$ and 0 otherwise. This measure is the true expected response, under the simulation model, implied by the estimated dosing rule. Comparing this to the target response will allow us to measure the accuracy of our dosing rule. For each q=1,…,Q and $K=1,\ldots ,K_{\max}$ we find $Y_{qK,\text{diff}}$ , the absolute difference between ${\hat{E}}^{\left(m\right)}\left[Y_{qK}\right]$ and $Y^{*}$ averaged over the 1000 simulated data sets

$$Y_{qK,\text{diff}}=\frac{1}{M}\sum_{m=1}^{M}\left|\hat{E}\left[Y_{qK}^{\left(m\right)}\right]-Y^{*}\right|$$

This measure can be interpreted as the accuracy of the *K*-group optimal dosing rule at age *A_q_*. As with Section 6.1, we calculate the integral of $Y_{qK,\text{diff}}$ over age using Simpson’s integration. This measure gives an overall measure of the accuracy of the *K*-group optimal dosing rule and allows us to evaluate how close the true expected response (derived from the simulation model) is to the target response when children are dosed according to the estimated optimal dosing rule. We also consider how many of the simulated data sets would lead us to select a dosing rule with $K^{*}=1,\ldots ,K_{\max}$ groups, in order to evaluate the typical complexity of optimal dosing rules and how this varies with the extent of differences between E–R model parameters across age groups.

## 7 Results

**Figure 3.**
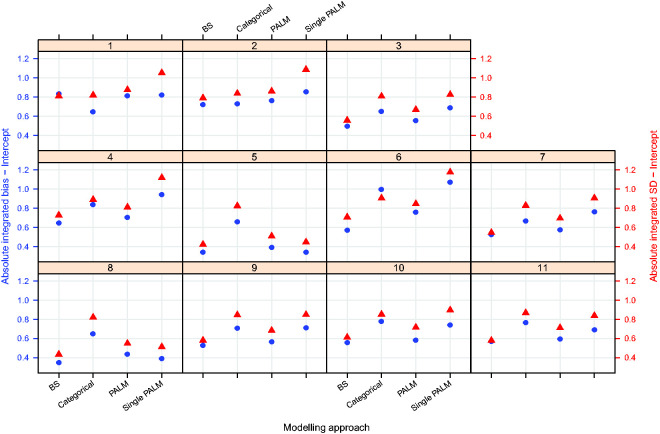
Integrated absolute bias (blue circles) and integrated empirical SD (red triangles) for the E–R model intercept. On the horizontal axis, ‘BS’ refers to the Bayesian penalised B-splines approach, ‘Categorical’ the linear model adjusted for a categorical age covariate, and ‘PALM’ and ‘singlePALM’ label the bootstrapped PALM tree approach and single PALM tree, respectively.

**Figure 4. fig4-0962280220903751:**
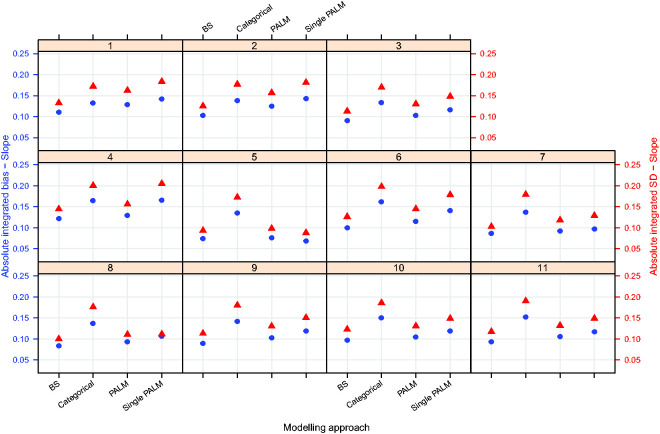
Integrated absolute bias (blue circles) and integrated empirical SD (red triangles) for the slope of the E–R model. On the horizontal axis, ‘BS’ refers to the Bayesian penalised B-splines approach, ‘Categorical’ the linear model adjusting for a categorical age covariate, and ‘PALM’ and ‘singlePALM’ label the bootstrapped PALM tree approach and single PALM tree, respectively.

Figures 3 [Fig fig4-0962280220903751]to 5 plot the integrated absolute bias and integrated empirical SD of E–R model parameter estimators for each modelling approach in each simulation scenario. For estimates obtained fitting Bayesian penalised B-splines, bootstrapped PALM trees, a single PALM tree and the linear model with categorical age covariate, Supplementary Tables S2 to S5, in Supplementary Appendix C, present the integrated AAB, empirical SD (as shown in Figures 3 [Fig fig4-0962280220903751]to 5) and empirical MSE (not included in the paper) of the estimated intercepts, slopes and expected response.

**Figure 5.**
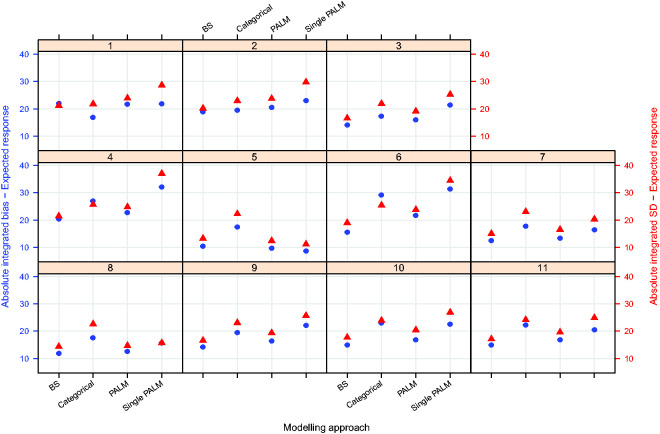
Integrated absolute bias (blue circles) and integrated empirical SD (red triangles) for the expected response. On the horizontal axis, ‘BS’ refers to the Bayesian penalised B-splines approach, ‘Categorical’ the linear model adjusting for a categorical age covariate, and ‘PALM’ and ‘singlePALM’ label the bootstrapped PALM tree approach and single PALM tree, respectively.

Comparing different modelling approaches within a scenario, Figures 3 [Fig fig4-0962280220903751]to 5 suggest that, in general, estimates of the functional relationship between the E–R model intercept and slope parameters obtained via Bayesian penalised B-splines are more accurate than estimates obtained using bootstrapped PALM trees. The single PALM tree fit is outperformed by the bootstrapped PALM tree approach in terms of both integrated absolute bias and empirical SD across most scenarios and both parameters, suggesting that bootstrapping is a refinement to the single PALM tree approach. As would be expected, the categorical covariate fit performs best in terms of accuracy and precision in scenario 1, where age groups are most distinct and follow the categories suggested by the ICH E11 guidance, excluding pre-term newborns.

**Figure 6.**
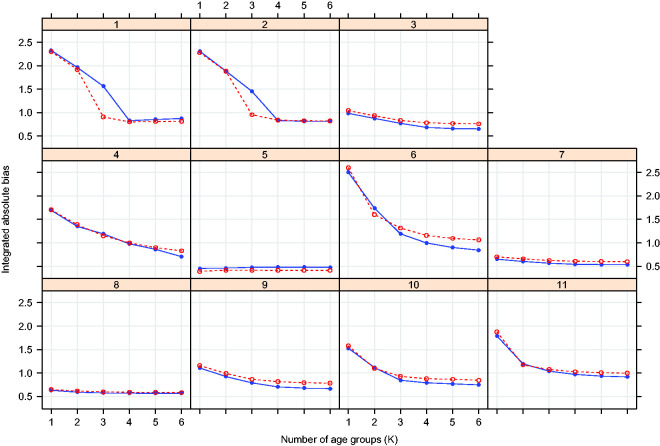
Integrated absolute difference between the target response and true expected response when children are dosed according to the *K* group optimal dosing rule. Results are shown for dosing rules obtained modelling the E–R relationship using Bayesian penalised B-splines (solid blue line) and bootstrapped PALM trees (dashed red line).

Figure 6 compares the performance of dosing rules minimising *G_K_* under different values of *K*, derived from E–R models fitted using different modelling approaches. As the linear model adjusting for a categorical age covariate has fixed age groups and the single PALM tree approach estimates specific age groupings, results for optimised dosing rules are only presented for the Bayesian penalised B-splines and bootstrapped PALM tree approaches. Figure 6 shows that overall both Bayesian penalised B-splines and bootstrapped PALM trees define *K*-group dosing rules with a similar performance in terms of getting the expected response close to the target response under the simulation model. In most scenarios, there comes a point at which there is little to be gained in terms of accuracy by refining the dosing rule further by allowing for additional age groups. As *K* increases, typically either the true expected response (under the simulation model and implied by the estimated dosing rule) better matches the target response or differences between the performances of the *K*-group dosing rules for ether modelling approach diminish.

**Figure 7.**
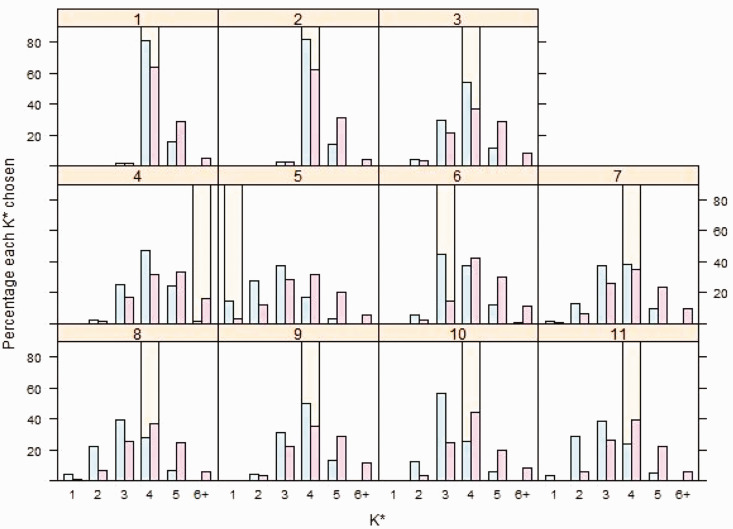
Percentage of 1000 simulations in which $K^{*}$ , the optimal number of age groups in the dosing rule, takes each value shown. $K^{*}$ is selected according to the algorithm described in Section 5.2 for Bayesian penalised B-spline (blue) and bootstrapped PALM tree (pink) approaches. The values of $K^{*}$ chosen by applying the algorithm in Section 5.2 to the true underlying E–R relationships in each scenario are shown by the yellow bars.

Figure 7 shows the percentage of simulations where the global optimum dosing rule comprises $K^{*}$ age groups for various values of $K^{*}$ . It is important to note, however, that the dosing rule age groups determined using the algorithm defined in Section 5.2 may not necessarily be identical to the true underlying E–R age groups, as if there are large differences in expected response between underlying E–R age groups, a better fit may be achieved by dosing rules splitting age groups around big changes and combining age groups with smaller changes. However, it is interesting to explore the values of $K^{*}$ defining the global optimal dosing rules to assess their complexity. Additionally, the complexity of the derived dosing rules will depend on the quantitative threshold used to identify $K^{*}$ described in Section 6.2; with a different threshold, *c*, dosing rules with different $K^{*}$ may be selected as optimal. We find optimal dosing rules minimising *G* to be cautious, forming slightly more age groups than the underlying E–R age groups.

Focusing on Bayesian penalised B-splines, we see from Figure 7 that in scenario 1, where larger differences are present in the underlying E–R model parameters, the large majority (81.6%) of simulated data sets would lead to the investigator selecting a global optimum dosing rule with $K^{*}=4$ , as would a smaller majority (54%) of data sets in scenario 3. This suggests that when underlying E–R relationships across age groups become less distinct, dosing rules with smaller $K^{*}$ are selected. In scenario 4, the majority of simulated data sets would lead to the investigator selecting global optimum dosing rules with $K^{*}=4$ , although there is a trend to larger $K^{*}$ compared with other scenarios. In scenario 5, where underlying E–R model parameters do not depend on age, a higher percentage of data sets lead to the selection of a dosing rule defined by a smaller $K^{*}$ .

Similar patterns are seen for the bootstrapped PALM trees approach in Figure 7. It seems that both bootstrapped PALM trees and the Bayesian penalised B-splines approach are capable of identification of dosing rules with multiple age groups when differences in the underlying E–R relationships across age groups are large, but fewer are identified as differences diminish. For the single PALM tree fit, for scenarios where larger differences are present in the underlying E–R model parameters, as in scenarios 1 and 2, a single PALM tree often identifies dosing rules with four groups; 96.9% and 94.6% would choose four groups, respectively. In not one scenario did a single PALM tree select a dosing rule with $K^{*}>4$ .

## 8 Extension to Emax model

**Figure 8.**
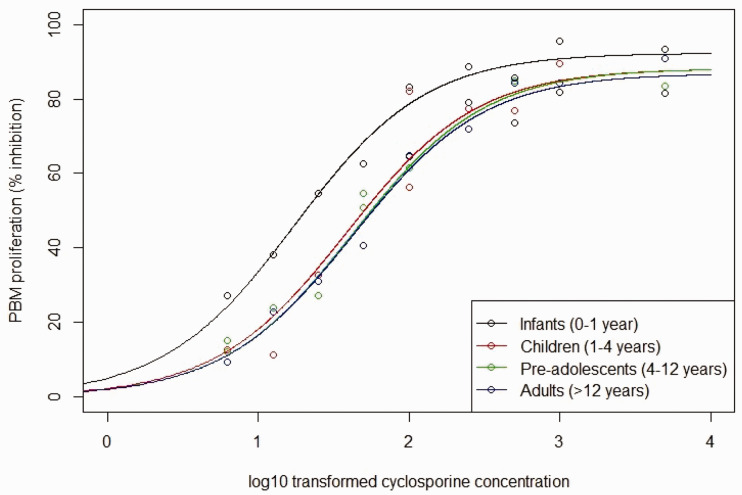
Fitted curves of the relationship between log base-10 transformed cyclosporine concentrations and PBM proliferation based on frequentist two-parameter Emax model fit for each of the four age groups considered. Fitted curves are the solid lines and the points are simulated data. (a) Emax. (b) EC50.

We consider a simulated example informed by the data presented in Marshall and Kearns,^37^ who modelled the relationship between cyclosporine concentration and in vitro inhibitory effect on peripheral blood monocyte (PBM) proliferation as a sigmoid Emax curve (2). We simulate responses for 41 subjects assigned to one of four age groups: 10 infants (0-1 year); 12 children (1-4 years); 9 pre-adolescents (4-12 years); and 10 adults (12-18 years). Data are generated such that for each of the following concentrations of cyclosporine (6.25; 12.5; 25; 50; 100; 250; 500; 1000 and 5000 ng/mL) a patient was recruited from each age group and the remaining patients in each age group (one infant; three children; one adult) were randomly assigned a concentration from this set. Within an age group, patients’ ages are assumed to follow a uniform distribution. In a deviation from Marshall and Kearns,^37^ patient responses are simulated according to a hyperbolic Emax model (setting δ(A)≡1 ), although we follow the original publication to force a zero intercept ( $\gamma_{0}(A)\equiv 0$ ). Patient responses are simulated setting the remaining EC50 and Emax model parameters equal to the age group-specific parameter estimates provided by Marshall and Kearns,^37^ and we assume a normally distributed random error with mean zero and variance 15. We restrict attention to a hyperbolic Emax model because estimates of age group-specific Hill parameters are not reported by Marshall and Kearns. Using these simulated data, we fitted a two-parameter Emax model separately to each age group. Figure 8 shows the four fitted curves.

### 8.1 Bayesian penalised B-splines

We implement the Bayesian penalised B-splines model by running three Markov chains using a thinning rate of 3 and 9000 iterations, 4500 of which are discarded as burn-in samples. We adopt the first-order random walk prior defined in Section 4.3 for the penalisation. We found a great deal of sensitivity, in terms of convergence, to the choice of prior for the standard deviation parameters of the random walk priors on the B-spline coefficients of the Emax and EC50 parameters. This sensitivity was found when using the Inverse-Gamma priors as used in Section 6. We would advise caution and appropriate checks to ensure posterior results are reliable. One should check a priori the plausible range of values for these standard deviations, which would depend on the magnitude of the Emax and EC50 parameters. Gamma (2,1/A) priors, with *A* large (such as *A *=* *10) are recommended by Chung et al.^38^ and the Stan user guide^39^ as boundary-avoiding priors in hierarchical models for hierarchical standard deviations. Placing Gamma (2,0.1) priors on the random walk prior standard deviations allowed the two-parameter Emax model to fit well to the simulated data shown in Figure 8, with the chains converging with Gelman–Rubin convergence diagnostic < 1.011 for all parameters.

**Figure 9.**
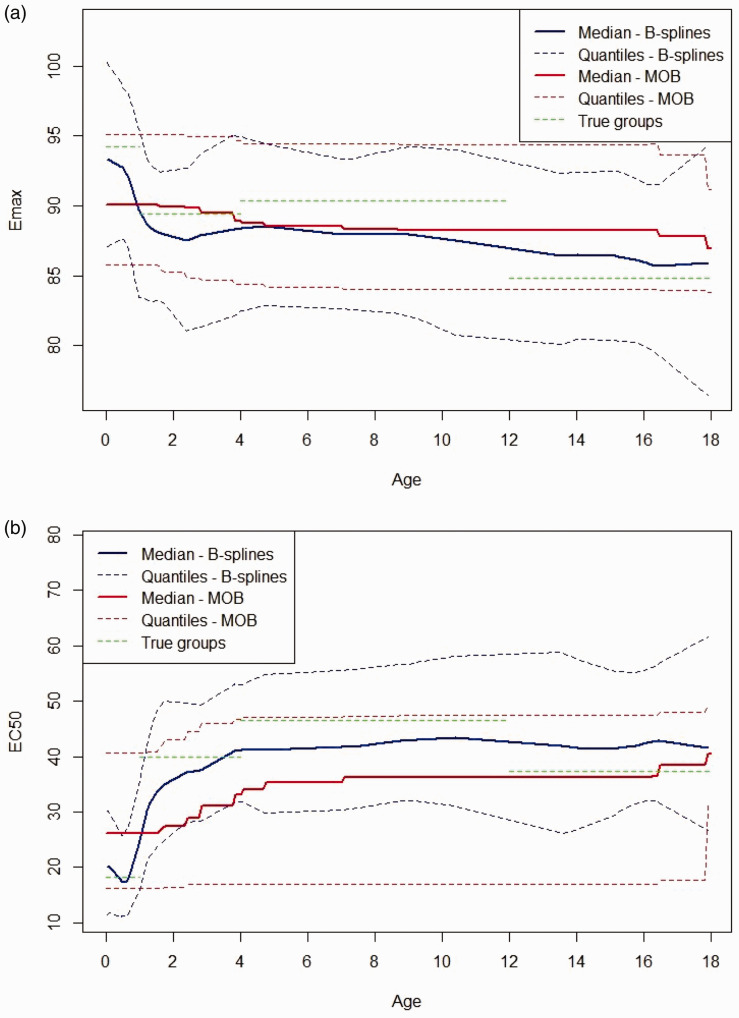
Plots of the Bayesian penalised B-spline and bootstrapped MOB fits of (a) the Emax parameter and (b) the EC50 parameter. The median of each parameter, with 2.5th and 97.5th quantiles, over the 1000 simulated bootstrap samples and true parameter values reported by Marshall and Kearns^37^ given by the green dotted lines are also shown. (a) B-splines. (b) MOB. MOB: model-based recursive partitioning.

**Figure 10.**
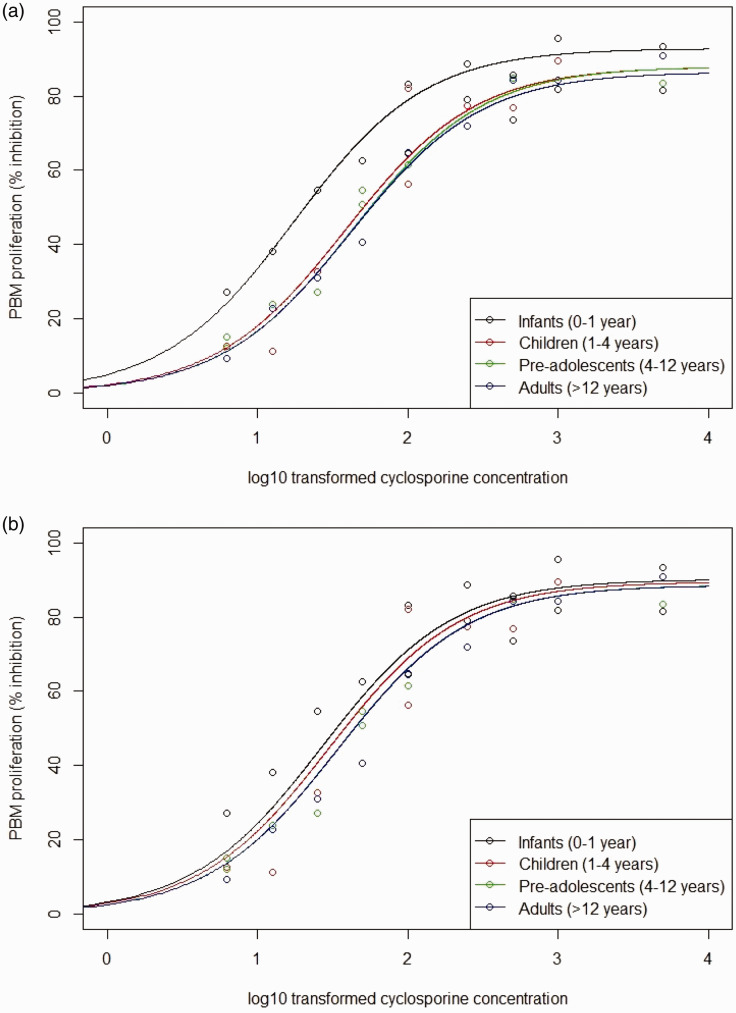
Fitted relationships between log base-10 transformed cyclosporine concentrations and PBM proliferation based on parameter estimates for the four age groups obtained with (a) the Bayesian penalised B-spline approach and (b) the bootstrapped MOB approach.

Figure 9 shows the fitted Bayesian penalised B-spline for the Emax and EC50 parameters over age, showing the median, 2.5th and 97.5th quantiles. The fitted B-splines for both the EC50 and Emax parameters seem to follow closely to the true underlying parameter values and, as can be seen from Figure 10(a), the underlying E–R relationships are accurately estimated. Figure 10(a) plots fitted expected response against concentration in each of the four age groups. The fitted expected response is calculated by setting the Emax and EC50 to values obtained by evaluating the Emax and EC50 fitted B-splines at the mid-points of each age group.

### 8.2 Bootstrapped MOB

To implement the bootstrapped MOB approach, we used the ‘mob’ function in R with a two-parameter Emax model. Otherwise, the approach proceeds exactly as the bootstrapped PALM trees approach described in Section 4.2. To incorporate a two-parameter Emax model in the ‘mob’ function, we built on code provided by Thomas et al.,^14^ using the ‘nls’ function in R^26^ to specify the two-parameter Emax model.

Figure 9 shows the fitted bootstrapped MOB for the Emax and EC50 parameters over age, showing the median, 2.5th and 97.5th quantiles over the bootstrapped samples. The fitted Emax and EC50 parameters do change with age. However, they are both quite far from the true underlying values. When looking at Figure 10(b), we see that the model still fits fairly well to the general shape of the data. However, Figure 10(b) highlights that there is worse separation between the fitted E–R curves for different age groups across the whole concentration range when using the bootstrapped MOB approach as compared with the Bayesian penalised B-splines.

### 8.3 Deriving dosing rules

**Figure 11.**
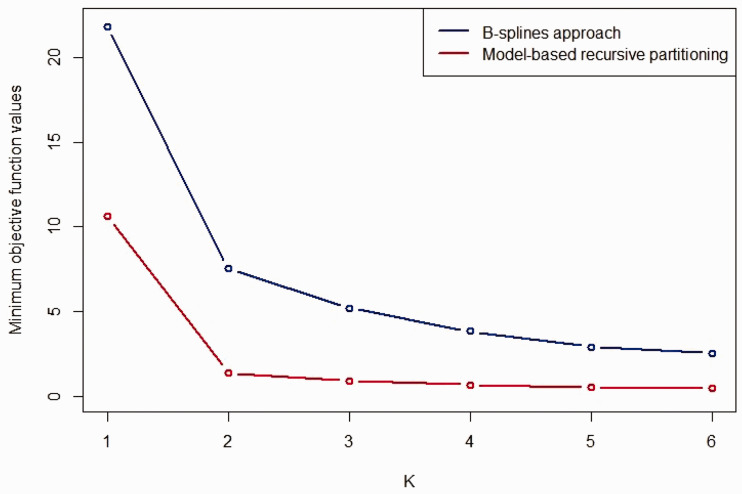
Plot of the objective function values from the optimisation procedure used to identify age groups for the Bayesian penalised B-splines approach (blue line) and bootstrapped MOB (red line).

Following the procedure to derive optimal dosing rules described in Section 5, Figure 11 provides a plot of the objective function values for rules based on both the Bayesian penalised B-splines and bootstrapped MOB approaches. Overall, the bootstrapped MOB approach achieves lower objective function values. For both approaches, two age groups would almost certainly be recommended by visual inspection.

For two age groups, the optimal age groups defining the bootstrapped MOB dosing rule would be 0 to 3.33 years and 3.33 to 18 years, with target exposures of 191.95 and 294.87, respectively. The optimal age groups defining the Bayesian penalised B-splines dosing rule would be 0 to 0.84 years and 0.84 to 18 years, with target exposures of 110.36 and 446.04, respectively. It is interesting to note how different the dosing rules are for these two methods: the bootstrapped MOB rule stipulates a wider youngest age group, with larger target exposure levels than the Bayesian penalised B-splines rule. However, overall the bootstrapped MOB dosing rule has a lower maximum target exposure than the Bayesian penalised B-splines dosing rule. This seems to be indicative of the larger differences between the age group-specific E–R relationships found using the Bayesian penalised B-splines approach.

## 9 Discussion

In this paper, we have considered several approaches to estimating if and how E–R model parameters change with age in order to determine practical dosing rules for distinct paediatric age groups. Our approaches concentrate on the relationship between exposure and response, deriving target exposures for age groups. These target exposures can then be used to identify dosing rules based on a separate relationship between dose and exposure. We do not develop PK models relating dose and exposure in this paper, although many methods exist to do this.^40^ In other therapeutic areas, non-monotonic changes in E–R model parameters over some age intervals may be plausible. Evaluating the performance of our methods in these scenarios is outside the scope of this paper, but is something that could be investigated in future research.

We derive the target exposures for each age group by taking the age group mid-point and finding the exposure level at which the expected response would be equal to the target response. In reality, this may not actually be the optimal exposure level over the whole age group. Rather than using the exposure appropriate for the age group midpoint, an alternative approach for deriving a more accurate target exposure would be the following: within an age group, target the single exposure level which minimises the total absolute difference between the expected response associated with the target exposure and the target response, integrating over the age group. This approach is computationally more demanding making it unsuitable for use in our simulation study, but can be quickly implemented for one data set in practice.

Results of our simulations with linear E–R models suggest that the Bayesian penalised B-splines and bootstrapped PALM tree approaches perform similarly in terms of estimating changes in E–R model parameters over age, though the integrated absolute bias and empirical SD is consistently lower in the Bayesian penalised B-splines approach. Plots of the absolute difference between the true expected response implied by proposed target exposures and the target response also suggest that for most scenarios both approaches perform similarly, though in some scenarios Bayesian penalised B-splines perform better than bootstrapped PALM trees, and vice versa. In fact, the Bayesian penalised B-splines approach appears to outperform all other approaches in most scenarios; only the approach using categorical covariates sometimes has lower integrated absolute bias, and even then, only in scenarios where the true underlying E–R models contain four age groups matching ICH E11 guidance (as is assumed in the categorical covariates approach).

## Supplemental Material

SMM903751 Supplemental Material1 - Supplemental material for Exposure–response modelling approaches for determining optimal dosing rules in children Supplemental material, SMM903751 Supplemental Material1 for Exposure–response modelling approaches for determining optimal dosing rules in children by Ian Wadsworth, Lisa V Hampson, Björn Bornkamp and Thomas Jaki in Statistical Methods in Medical Research

## Appendix 1: Simulation scenarios in detail

In terms of how the E–R model parameters change over age, we consider 11 scenarios for data generation:

1. Step function relates how E–R model parameters change over age, following ICH guidance document age groupings with substantial differences between E–R model parameter values in each age group;
2. E–R model parameters have a less steep linear transition between age groups, with parameter values and age groups the same as scenario 1;
3. E–R model parameter values have same change over age as in scenario 2, though now age groups parameter values are more similar between age groups to more closely resemble what might be observed in reality;
4. Step function relates how E–R model parameters change over age, as in scenario 1, but now there is a deviation from the ICH age groups so that there are more distinct age groups amongst younger children, following NICHD groupings;
5. E–R model parameters constant over all age groups;
6. E–R model parameters have a constant linear decrease over the whole age range;
7. Intercept term as in scenario 3, slope term constant over age as in scenario 6;
8. Intercept term constant over age as in scenario 6, slope term as in scenario 3;
9. Same as scenario 3, though now the true age groups will be 0 to 6 months, 6 months to 3 years, 3 years to 11 years, 11 years to 18 years;
10. Same as scenario 3, though now the true age groups will be 0 to 2 year, 2 year to 6 years, 6 years to 14 years, 14 years to 18 years;
11. Same as scenario 3, though now the true age groups will be 0 to 8 years, 8 years to 12 years, 12 years to 16 years, 16 years to 18 years.
