# Supplementary material for: Exposure–response modelling approaches for determining optimal dosing rules in children
Source: Stat Methods Med Res. 2020 Feb 13;29(9):2583–602. doi: 10.1177/0962280220903751 (PMC7528535; doi:10.1177/0962280220903751)
Supplement: SMM903751 Supplemental Material1 - Supplemental material for Exposure–response modelling approaches for determining optimal dosing rules in children [file SMM903751_Supplemental_Material1.pdf]

Online supplementary material: Exposure-response modelling approaches for determining optimal dosing rules in children

Ian Wadsworth, Lisa V. Hampson, Björn Bornkamp, Thomas Jaki

1 Supplementary Appendix A: Worked example of methods

This supplementary appendix aims to give an illustration of the output that would be seen from fitting each of the methods in Section 4 to a single set of simulated data. For each of the approaches we estimate the relationship between intercept or slope and age. For $i = 1, \ldots, 100$ subjects, we simulate the response as:

$$Y_i = \begin{cases} 5.1 - 0.010C_i + \epsilon_i, & \text{for } A_i \in (0, 4] \\ 4.8 - 0.035C_i + \epsilon_i, & \text{for } A_i \in (4, 10] \\ 4.4 - 0.075C_i + \epsilon_i, & \text{for } A_i \in (10, 14] \\ 3.9 - 0.125C_i + \epsilon_i, & \text{for } A_i \in (14, 18] \end{cases}$$

where the C_i exposure values are simulated as in Section 6 of the paper following Wadsworth et al. [1] and the ϵ_i are random errors simulated from a normal distribution with mean 0 and variance 0.02. The A_i age values are simulated from four Uniform distributions such that there are 25 subjects in each of four age groups: 0 to 4 years; 4 to 10 years; 10 to 14 years; and 14 to 18 years.

First, the linear model with categorical covariates as shown in Section 4.1 is fitted to the simulated example data. Using the true age groups to define $A_{1i}, \ldots, A_{Hi}$ (the age groups used for the categorical covariates), the following intercepts and slopes are estimated in turn for each of the four age groups: intercept estimates are 5.13, 4.80, 4.42 and 3.84; and slope estimates are -0.010, -0.031, -0.080 and -0.120.

Now, we fit a single PALM tree model as described in Section 4.2. Figure S1 shows the results of a PALM tree fitted to simulated data; this is standard output from the 'partykit' package [2, 3]. Four nodes (here, age groups) have been found in the following age groupings: 0 to 3.89 years; 3.89 to 9.94 years; 9.94 to 14.00 years; and 14.00 to 18 years. Other than 0 and 18 (fixed based on the paediatric population), the age group limits are observed age values from the data; were there an age data point less than 4, but closer to 4 than 3.89, this age boundary could be even closer to the truth. Regardless, these age groups are very

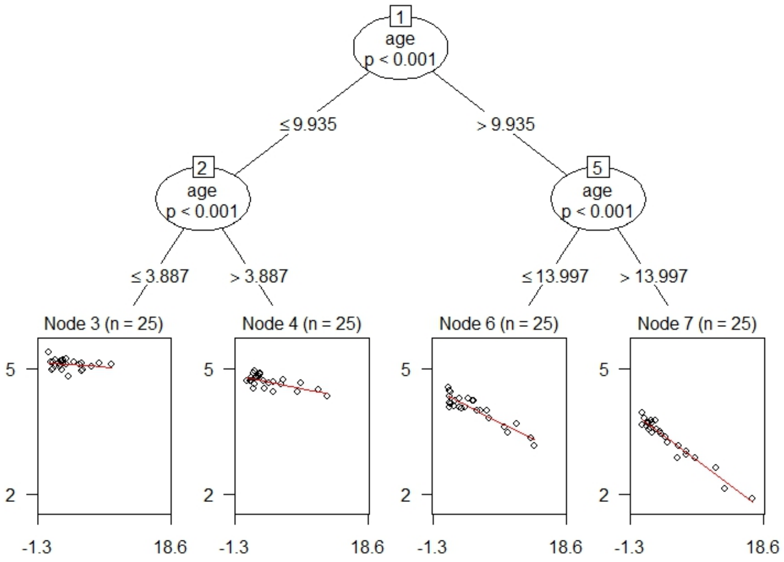

Figure S1: Example to demonstrate the structure of a single PALM tree fitted to simulated data, produced from the 'partykit' package [2, 3].

close to the true age groups and estimate the underlying PK-PD parameters well also. For each age group in turn, the intercepts are 5.13, 4.80, 4.42 and 3.84 and the slopes are -0.010, -0.031, -0.080 and -0.120. For this data, this model gives identical estimates to the linear model with categorical covariates, to six decimal places.

We then extend to the bootstrapped PALM trees also described in Section 4.2. Figure S2 presents plots of the bootstrapped PALM fits of intercept and slope parameters over age constructed by following the approach given in Section 4.2. We plot the relationship between intercept or slope against age using the bootstrap averaged median, 2.5th and 97.5th quantiles at a continuum of ages from 0 to 18 years, also highlighting the true underlying intercept/slope values by green dashed lines. The 2.5th and 97.5th quantile lines are asymmetric as the the distribution (over the bootstrap samples) of the intercept / slope values is asymmetric at many age values from 0 to 18.

Next, we apply the B-splines approach described in Section 4.3. Figure S3 presents plots of the median, 2.5th and 97.5th quantiles of the posterior distributions of the intercept/slope parameters at each A_i from the MCMC output of the B-spline model fit, also highlighting the true underlying intercept/slope values by green dashed lines.

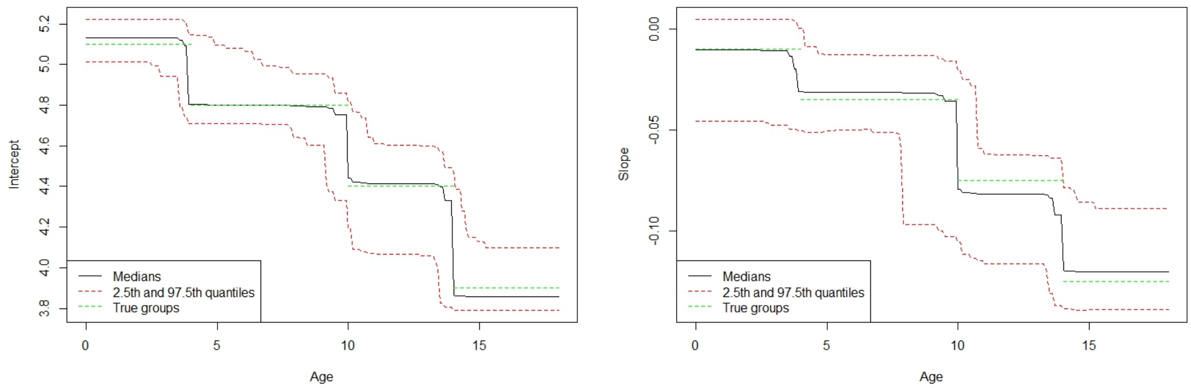

(a) (b)

Figure S2: (a) Plot of the the intercept parameter over age from the bootstrapped PALM fit to the simulated example data, showing the median intercept, with 2.5th and 97.5th quantiles, over the 1000 simulated bootstrap samples and true parameter values given by the green dotted lines. (b) Plot of the the slope parameter over age from the bootstrapped PALM fit to the simulated example data, showing the median slope, with 2.5th and 97.5th quantiles, over the 1000 simulated bootstrap samples and true parameter values given by the green dotted lines.

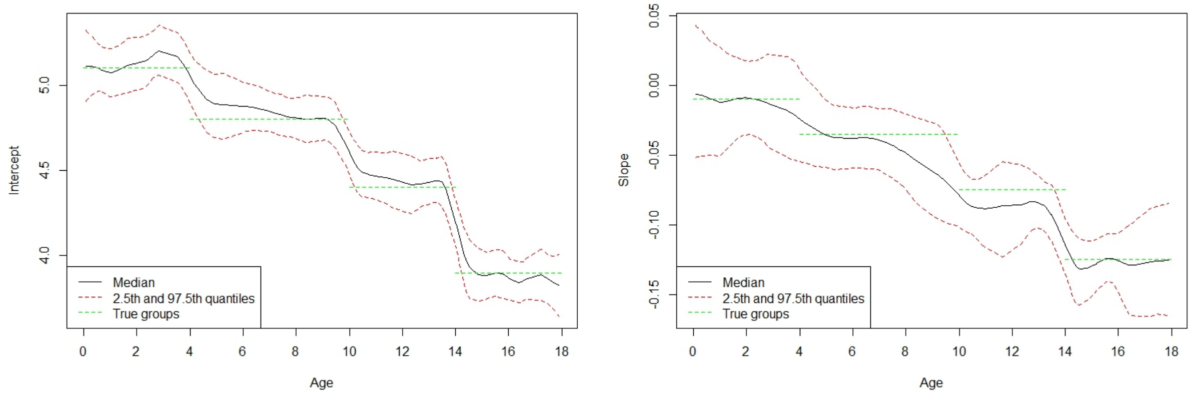

(a) (b)

Figure S3: (a) Plot of the the intercept parameter over age from the B-spline fit to the simulated example data, showing the median intercept with 2.5th and 97.5th quantiles and true parameter values given by the green dotted lines. (b) Plot of the the slope parameter over age from the B-spline fit to the simulated example data, showing the median slope with 2.5th and 97.5th quantiles and true parameter values given by the green dotted lines.

2 Supplementary Appendix B: Inclusion of additional covariate

For all scenarios given in the paper, the response has been modelled as in equation (1) without additional covariates $x_{1i}, \ldots, x_{Pi}$. In this supplementary appendix, the data are generated such that there is a relationship between response and an additional covariate.

Assume we have data on body weight, x_w, which is modelled as a linear function of age; the linear relationship we use, $x_w = 3A + 7$, is based on the use of weight estimation in paediatrics (1 to 13 years, inclusive) suggested by Luscombe et al. [4], though other suggested weight/age relationships exist. We assume that, like age, this covariate has an effect on response. However, as body weight here is largely explained by age it feels more natural to regress body weight against age and to consider the effect of the fitted residuals, r_w, in the model, essentially, what effect of body weight on response remains after adjusting for age. We simulate the response in this example by having the body weight residuals, r_w (which have a standard normal distribution), included in the simulation model as follows:

$$Y_i = \gamma_0(A_i) + 0.4r_w + \gamma_C(A_i)C_i + \epsilon_i. \quad (1)$$

When fitting the model, we consider two approaches: one approach where body weight has been observed and is included in the model; and a variation where the effect of the body weight residuals still exists, though body weight is now an unobserved covariate and not included in the model. The directed acyclic graph shown in Figure S5 aims to visualise this causal relationship. For this second modelling approach, we seek to identify how the methods cope when there is an effect that we are unable to observe and control for. We will therefore fit the models in Section 4 to this scenario, modelling the PD response in two ways.

Comparing the panels in Figures S6 representing the supplementary scenario with and without r_w, it is clear that when the additional covariate is included in the simulation model, but not included in the analysis model, all approaches do not perform as well at estimating the underlying relationship between age and the exposure-response model slope or intercept parameters. However, the B-splines approach again seems to perform better than the other approaches in terms of accuracy and precision.

Figure S7 shows that when the additional covariate is not included in the analysis model, the accuracy of the K-group optimal dosing rule is lower and the true expected response (derived from the simulation model when children are dosed according to the estimated optimal dosing rule) is further from the target response, for both the bootstrapped PALM trees and Bayesian penalised B-splines approaches. However, the Bayesian penalised B-splines approach does seem to provide better accuracy than the bootstrapped PALM trees. Figure S8 shows that when the additional covariate is included in the analysis model, the majority of simulated datasets would lead to the investigator selecting a global optimum dosing rule with $K^* = 4$, especially for the Bayesian penalised B-splines approach.

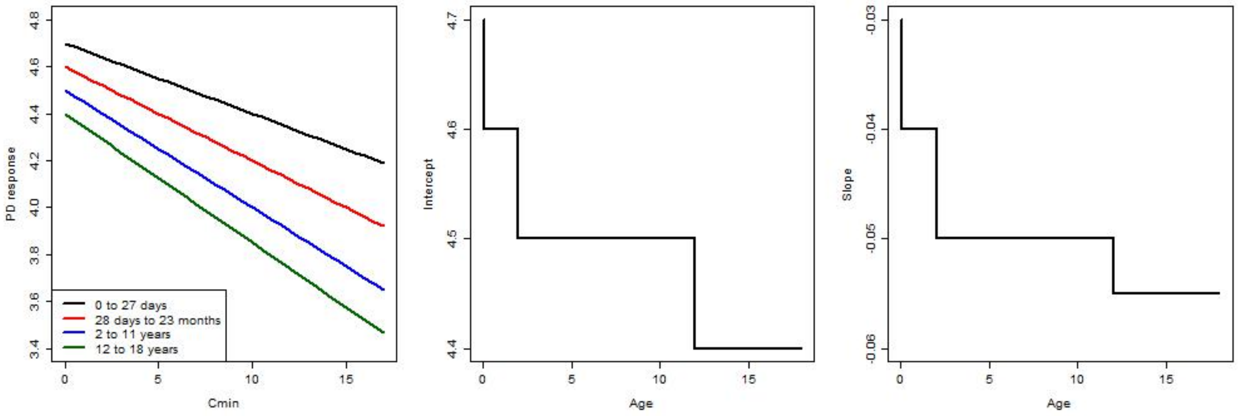

Figure S4: Plots for the supplementary scenario showing (1) the true underlying E-R relationship; (2) how the intercept of the E-R model changes with age; (3) how the slope of the E-R model changes with age.

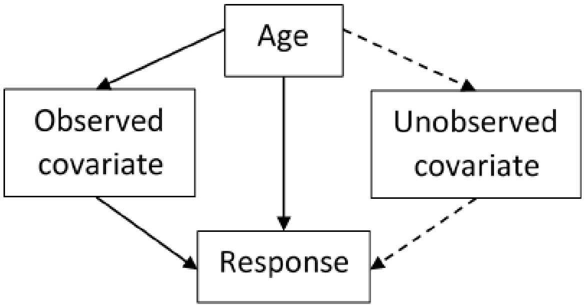

Figure S5: Directed acyclic graph illustrating the causal relationships between age, observed/unobserved covariates and response.

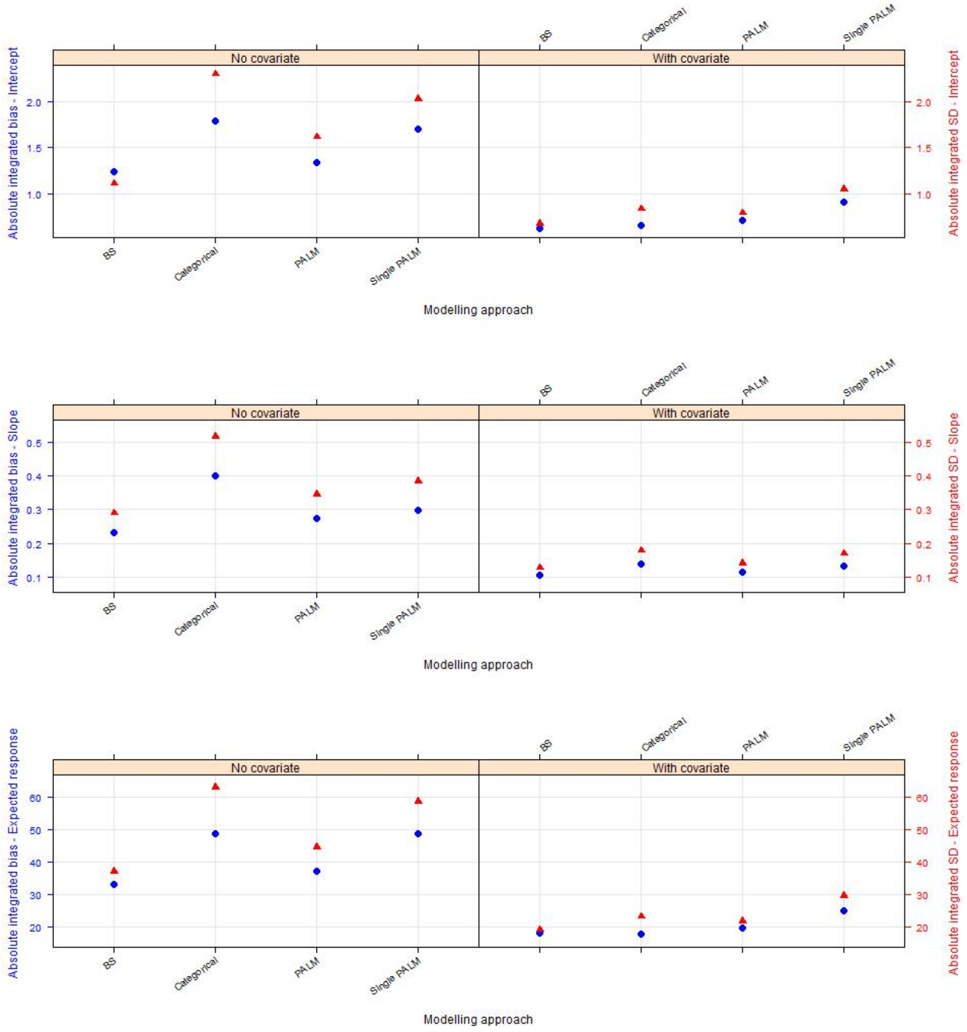

Figure S6: Integrated absolute bias (blue circles) and integrated empirical SD (red triangles) for (1) E-R model intercept; (2) E-R model slope; (3) expected response. On the horizontal axis, 'BS' refers to the Bayesian penalised B-splines approach, 'Categorical' the linear model adjusted for a categorical age covariate, and 'PALM' and 'singlePALM' label the bootstrapped PALM tree approach and single PALM tree, respectively. Panels display the supplementary scenario with and without the additional covariate in the analysis model.

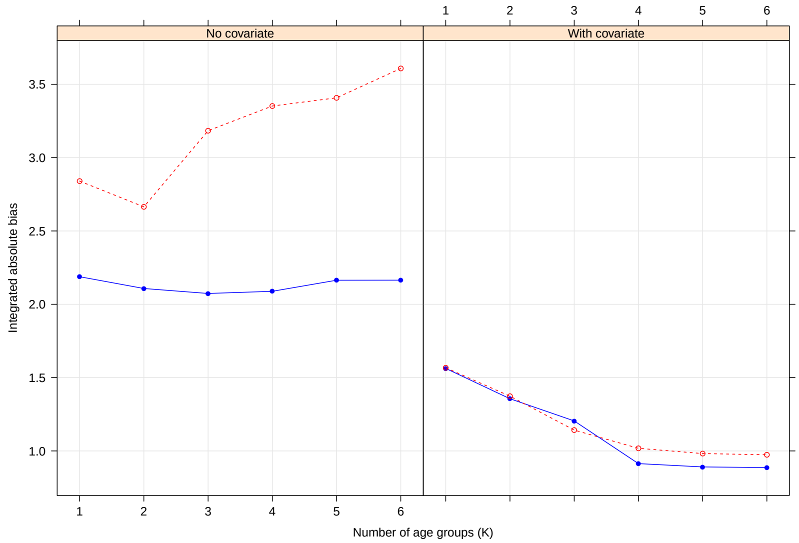

Figure S7: Integrated absolute difference between the target response and true expected response when children are dosed according to the K group optimal dosing rule, for the supplementary scenario. Results are shown for dosing rules obtained modelling the E-R relationship using Bayesian penalised B-splines (solid blue line) and bootstrapped PALM trees (dashed red line).

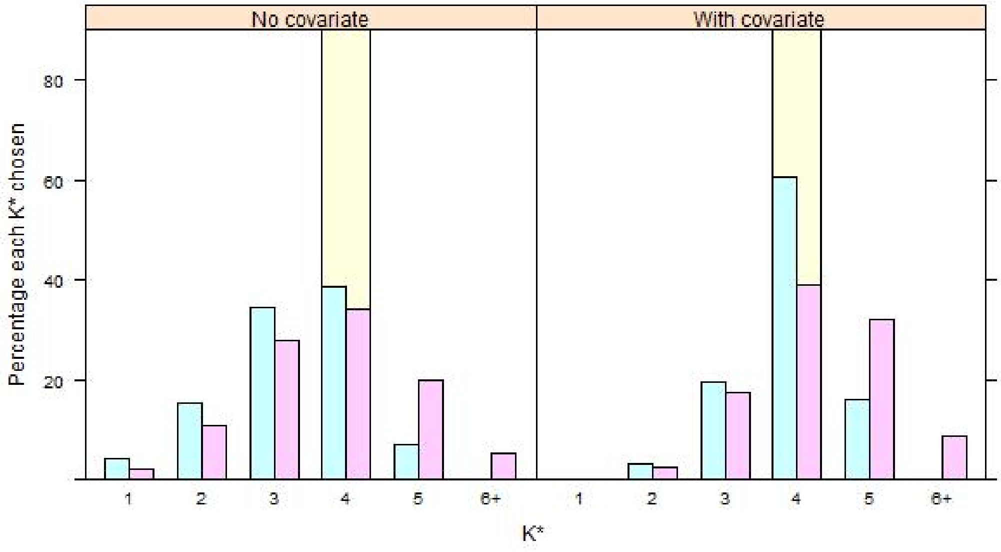

Figure S8: Percentage of 1000 simulations in which K^*, the optimal number of age groups in the dosing rule, takes each value shown. K^* is selected according to the algorithm described in Section 5.2 in the paper for Bayesian penalised B-spline (blue) and bootstrapped PALM tree (pink) approaches. The values of K^* chosen by applying the algorithm in Section 5.2 to the true underlying E-R relationships in the supplementary scenario are shown by the yellow bars.

3 Supplementary Appendix C: Supplementary tables

Table S1: True E-R model parameter values for age groupings in scenarios 1 to 8.

		Simulation scenario						
Parameter	Age group	1 & 2	3	4	5	6	7	8
γ_0	0 to 28 days	4.85	4.60	4.60	4.50	4.700 – 4.698	4.60	4.50
	28 days to 1 year	4.65	4.55	4.52	4.50	4.698 – 4.680	4.55	4.50
	1 to 2 years	4.65	4.55	4.44	4.50	4.680 – 4.660	4.55	4.50
	2 to 6 years	4.45	4.50	4.35	4.50	4.660 – 4.580	4.50	4.50
	6 to 12 years	4.45	4.50	4.27	4.50	4.580 – 4.460	4.50	4.50
	12 to 18 years	4.25	4.45	4.20	4.50	4.460 – 4.340	4.45	4.50
γ_E	0 to 28 days	-0.045	-0.040	-0.035	-0.050	-0.050 – -0.050	-0.050	-0.040
	28 days to 1 year	-0.055	-0.045	-0.045	-0.050	-0.050 – -0.052	-0.050	-0.045
	1 to 2 years	-0.055	-0.045	-0.055	-0.050	-0.052 – -0.053	-0.050	-0.045
	2 to 6 years	-0.065	-0.050	-0.065	-0.050	-0.053 – -0.059	-0.050	-0.050
	6 to 12 years	-0.065	-0.050	-0.075	-0.050	-0.059 – -0.068	-0.050	-0.050
	12 to 18 years	-0.075	-0.055	-0.085	-0.050	-0.068 – -0.077	-0.050	-0.055

Table S2: Average absolute bias, empirical standard deviation (ESD) and empirical mean squared error (EMSE) for the estimated B-spline intercept/slope over age curve, integrated over age for each scenario considered.

	Intercept			Slope			Expected response		
Scenario	Bias	ESD	EMSE	Bias	ESD	EMSE	Bias	ESD	EMSE
1	0.831	0.811	0.063	0.110	0.132	0.001	21.994	21.214	2.672
2	0.720	0.790	0.045	0.103	0.125	0.001	18.879	20.195	1.927
3	0.496	0.557	0.022	0.091	0.113	0.001	14.058	16.574	1.152
4	0.644	0.726	0.036	0.122	0.145	0.001	20.405	21.440	2.355
5	0.339	0.421	0.010	0.074	0.093	0.000	10.381	13.254	0.675
6	0.571	0.706	0.030	0.100	0.126	0.001	15.568	18.994	1.439
7	0.526	0.544	0.024	0.087	0.103	0.001	12.501	15.018	0.934
8	0.346	0.434	0.011	0.083	0.100	0.001	11.940	14.354	0.877
9	0.526	0.580	0.024	0.090	0.113	0.001	14.175	16.622	1.159
10	0.557	0.611	0.027	0.097	0.123	0.001	14.946	17.706	1.305
11	0.570	0.578	0.029	0.093	0.117	0.001	14.950	17.128	1.280

Table S3: Average absolute bias, empirical standard deviation (ESD) and empirical mean squared error (EMSE) for the estimated bootstrapped PALM intercept/slope over age curve, integrated over age for each scenario considered.

	Intercept			Slope			Expected response		
Scenario	Bias	ESD	EMSE	Bias	ESD	EMSE	Bias	ESD	EMSE
1	0.811	0.875	0.063	0.129	0.162	0.002	21.690	23.930	2.905
2	0.763	0.860	0.052	0.125	0.157	0.001	20.588	23.762	2.473
3	0.553	0.668	0.027	0.103	0.130	0.001	16.089	19.138	1.514
4	0.702	0.810	0.043	0.130	0.156	0.002	22.774	24.781	2.987
5	0.392	0.508	0.014	0.076	0.098	0.001	9.690	12.429	0.596
6	0.759	0.845	0.051	0.116	0.145	0.001	21.741	23.790	2.662
7	0.573	0.694	0.029	0.092	0.118	0.001	13.359	16.536	1.041
8	0.437	0.548	0.017	0.093	0.111	0.001	12.668	14.691	0.997
9	0.564	0.682	0.028	0.103	0.130	0.001	16.379	19.371	1.551
10	0.583	0.715	0.030	0.104	0.131	0.001	16.786	20.444	1.634
11	0.593	0.711	0.032	0.105	0.132	0.001	16.923	19.635	1.733

Table S4: Average absolute bias, empirical standard deviation (ESD) and empirical mean squared error (EMSE) for the estimated single PALM tree intercept/slope over age step function curve, integrated over age for each scenario considered.

	Intercept			Slope			Expected response		
Scenario	Bias	ESD	EMSE	Bias	ESD	EMSE	Bias	ESD	EMSE
1	0.821	1.051	0.076	0.142	0.183	0.002	21.921	28.674	3.548
2	0.856	1.086	0.073	0.143	0.181	0.002	23.066	29.816	3.434
3	0.689	0.825	0.041	0.116	0.148	0.001	21.454	25.290	2.514
4	0.942	1.117	0.078	0.166	0.205	0.003	32.076	37.016	5.766
5	0.340	0.446	0.011	0.069	0.088	0.0004	8.686	11.151	0.480
6	1.070	1.174	0.097	0.141	0.179	0.002	31.382	34.460	5.209
7	0.760	0.905	0.048	0.097	0.129	0.001	16.499	20.326	1.490
8	0.391	0.512	0.015	0.106	0.111	0.001	15.696	15.713	1.472
9	0.711	0.848	0.043	0.118	0.150	0.001	22.087	25.688	2.645
10	0.742	0.896	0.048	0.119	0.149	0.001	22.596	26.905	2.858
11	0.689	0.837	0.044	0.116	0.149	0.001	20.588	24.893	2.607

Table S5: Average absolute bias, empirical standard deviation (ESD) and empirical mean squared error (EMSE) for the estimated intercept/slope over age step function curve based on the categorical covariates model approach, integrated over age for each scenario considered.

	Intercept			Slope			Expected response		
Scenario	Bias	ESD	EMSE	Bias	ESD	EMSE	Bias	ESD	EMSE
1	0.645	0.819	0.037	0.133	0.172	0.002	16.881	21.832	1.868
2	0.729	0.837	0.048	0.138	0.177	0.002	19.589	22.973	2.419
3	0.648	0.807	0.037	0.134	0.170	0.002	17.299	21.917	1.910
4	0.837	0.889	0.062	0.165	0.200	0.003	26.975	25.637	4.257
5	0.657	0.823	0.038	0.135	0.172	0.002	17.434	22.263	1.929
6	0.996	0.904	0.086	0.162	0.198	0.002	29.114	25.421	4.722
7	0.664	0.827	0.039	0.137	0.179	0.002	17.761	23.049	2.094
8	0.651	0.821	0.037	0.137	0.176	0.002	17.628	22.626	2.020
9	0.708	0.845	0.044	0.141	0.180	0.002	19.509	23.098	2.398
10	0.776	0.851	0.054	0.150	0.186	0.002	23.047	23.907	3.179
11	0.765	0.867	0.051	0.152	0.190	0.002	22.264	24.177	2.971

4 Supplementary Appendix D: Supplementary plot

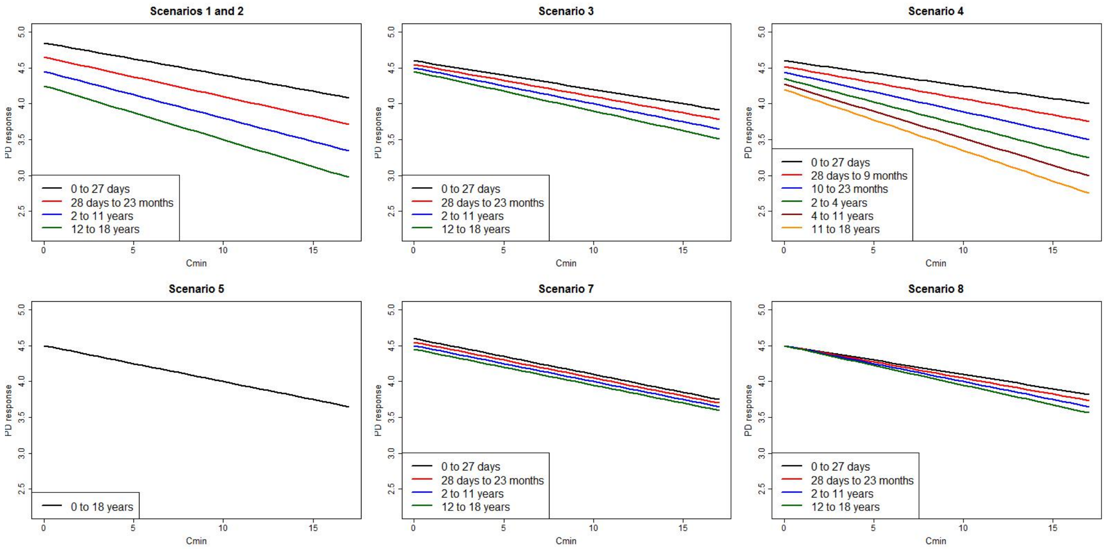

Figure S9: True underlying E-R relationships for scenarios 1 to 5, 7 and 8, as described in Appendix A of the paper.

References

[1] Ian Wadsworth, Lisa V Hampson, Thomas Jaki, Graeme J Sills, Anthony G Marson, and Richard Appleton. A quantitative framework to inform extrapolation decisions in children. *Under review*, 2018.

[2] Torsten Hothorn and Achim Zeileis. Partykit: A modular toolkit for recursive partytioning in R. *Journal of Machine Learning Research*, 16:3905–3909, 2015.

[3] Heidi Seibold, Torsten Hothorn, and Achim Zeileis. Generalised linear model trees with global additive effects. *arXiv preprint arXiv:1612.07498*, 2016.

[4] Mark D Luscombe, Ben D Owens, and Derek Burke. Weight estimation in paediatrics: a comparison of the APLS formula and the formula Weight= 3 (age)+ 7. *Emergency Medicine Journal*, 28(7):590–593, 2011.
